# Editorial: Multimorbidity in the context of neurodegenerative disorders

**DOI:** 10.3389/fnins.2022.1076486

**Published:** 2022-11-22

**Authors:** Rafael Linden, Maria Vassilaki, Emily J. Henderson, Devi Mohan

**Affiliations:** ^1^Instituto de Biofísica Carlos Chagas Filho, UFRJ, Rio de Janeiro, Brazil; ^2^Department of Quantitative Health Sciences, Mayo Clinic, Rochester, MN, United States; ^3^Population Health Sciences, Bristol Medical School, University of Bristol, Bristol, United Kingdom; ^4^Global Public Health, Jeffrey Cheah School of Medicine and Health Sciences, Monash University Malaysia, Subang Jaya, Malaysia

**Keywords:** multimorbidity, comorbidity, neurodegenerative disease, neurodegeneration, brain

The term *multimorbidity*, when applied to one person, is defined as the co-occurrence of two or more medical conditions, none of which qualifies as an *index* disorder (Boyd and Fortin, 2010; Skou et al., 2022). Such a combination is found among either transient or long-term physical, infectious, and/or mental health conditions. Mechanisms underlying the development of multimorbidity are complex; for example, Skou et al. summarized these mechanisms in three areas, i.e., relating to aging and inflammation; socioeconomic, psychosocial and behavioral determinants of health; and medication-related (Skou et al., 2022). Systemic multimorbidity is highly prevalent in older people (Marengoni et al., 2011; Skou et al., 2022), which is also of concern due to the progressive increase in human longevity (Oeppen, 2019).

Multimorbidity often includes Neurodegenerative Disorders (NDs), most of which are incapacitating, incurable and lack disease-modifying treatments. It may deceive early diagnosis, especially in low- and middle-income countries, crippled by a shortage of advanced imaging equipment (Hricak et al., 2021) and poor capacity. Nonetheless, an increasing number of studies worldwide report multimorbidity in patients previously diagnosed with a single neurological disease (Habek et al., 2020; Borm et al., 2022). Such findings may explain the failure of many clinical trials, which follow the usual rule that a prospective patient of a clinical trial be rendered ineligible in the presence of unrelated comorbidities (Weiss et al., 2014; Marrie et al., 2016; Unger et al., 2019). Remarkably, experiments with organotypic brain slices *in vitro* suggest that the progressive growth of an area of silent pathology in the brain may synergize with the leading edge of a distinct subliminal pathology, and such a combination may react in unexpected ways to certain drugs currently prescribed for either such NDs (Simões-Pires et al., 2021).

Near-exponential increase of publications in the past 30 years highlights the progressive awareness of Multi- and/or Comorbidity (https://pubmed.ncbi.nlm.nih.gov/?term=Multimorbidity+OR+Comorbidity&sort=date), inclusive among the hitherto small numbers of publications involving NDs (https://pubmed.ncbi.nlm.nih.gov/?term=%28%28Multimorbidity%29+OR+%28Comorbidity%29%29+AND+%28%28Neurodegeneration%29+OR+%28Neurodegenerative%29%29&sort=date), and both the interest and investment in this broad scientific field are recognized as of utmost importance (Navickas et al., 2016). Along these lines, the present Frontiers Research Topic aimed at novel approaches to multimorbidity in the context of Neurodegeneration, the contents of which are summarized here.

Santiago and Potashkin, from the Rosalind Franklin University of Medicine and Science, in Chicago, USA, offered an overview of the association of diabetes, cardiovascular disease, depression, and the gut microbiome, upon Alzheimer's disease (AD), and highlighted a variety of biological pathways involved in such comorbidities. Remarkably, inflammation stands out as a common dysregulated pathway shared by most of those comorbidities, and associated with increased risk of AD. In addition, certain drugs commonly prescribed for either diabetes or cardiovascular disease also show promising results in AD patients. The authors discussed the possible roles of both common dysregulated pathways, as well as genetic factors, in comorbidities associated with AD.

Luo et al., from the Medical Informatics Center at Peking University, in China, developed a novel multimorbidity index that incorporates disease combinations, as compared with individual diseases only, to predict 5-year mortality risk based on 13 chronic conditions among almost 12,000 community-dwelling older adults aged 65–84 years. The authors reported that the multimorbidity index incorporating disease combinations showed a better performance in predicting mortality among community-dwelling older adults. These findings strengthen the need to consider significant disease combinations to capture synergistic effects when evaluating multimorbidity in medical research and clinical practice.

Zhang et al., from Sichuan University, in China, tackled the problem that, despite abundant evidence that vascular risk factors (VRF) associate with cognitive impairment (CI), such association had not been studied in patients with multiple systems atrophy (MSA). The authors evaluated for CI a total of 658 patients with MSA only, MSA with predominant parkinsonism (MSA-P), and MSA with predominant cerebellar ataxia (MSA-C). All 3 groups had similar prevalences of CI, however, patients with more than one vascular risk factors were significantly more likely to have CI in both the MSA and MSA-P groups. Their findings that multiple VRF had a cumulative impact on CI in MSA patients highlights the need for comprehensive management of VRF in MSA.

El Idrissi and Alonso, from The City University of New York, USA, used a mouse model (PH-Tau-Tg) of Tau-induced neurodegeneration similar to that observed in Alzheimer disease, to test for interaction between pathological human Tau and Insulin signaling. The study showed that insulin signal transduction is altered in PH-Tau-Tg mice, and that injection of exogenous insulin reduces the excitability of cortical neuronal circuits. The authors propose that abnormal Tau may potentiate the toxic environment by interfering with the insulin signaling cascade and inducing insulin resistance in the brain of patients with Alzheimer's disease. The results favor the hypothesis that alterations in insulin signal transduction pathway may play a causative role in AD.

The article by the team of Stanisavljevic et al., of the University of Rhode Island, USA, aimed at testing the effects of Hypertension upon Cerebral Amyloid Angiopathy (CAA), a common comorbidity of Alzheimer's disease (AD). To that effect, the authors bred rTg-DI transgenic rats, a model of CAA, with spontaneously hypertensive stroke prone (SHR-SP) rats, producing bigenic rTg-DI/SHR-SP and non-transgenic SHR-SP littermates. The experiments showed, for example, that non-pharmacological hypertension in rTg-DI rats causes a redistribution of vascular amyloid and altered the size and distribution of thalamic occluded vessels. In addition, bigenic rTg-DI/SHR-SP rats provides a non-pharmacological model to further study hypertension and CAA as co-morbidities for CSVD and VCID.

Aiming at contributing to unveil the controversial relationship between vascular disease and Parkinson's disease, Ma et al. (a), from the Institute of Geriatric Medicine in Beijing, China, compared the total burden of cerebral small vessel disease (CSVD) in patients at either early or advanced Hoehn and Yahr (H&Y) scale stages of Parkinson's disease (PD), as well as in normal controls (NC). A total CSVD score was calculated for each participant, based on lacunes, high-grade white matter hyperintensities, enlarged perivascular spaces, and cerebral microbleeds. After adjusting for multiple variables, the data showed that higher H&Y stage correlated with increased total CSVD score. The overall analysis indicates that CSVD may play a critical role in patients with PD, and the total CSVD score is a potential neuroimaging marker for monitoring the progression of PD.

An article of the same group above, from the Institute of Geriatric Medicine in Beijing, China, used the diffusion tensor image analysis along the perivascular space (DTI-ALPS), to study the glymphatic system activity in patients with either early or late Parkinson's disease (PD) as compared with normal controls [NCs; Ma et al. (b)]. Patients with late, but no early, PD had lower ALPS index than NCs. Together with other results, the authors concluded that impairment of the glymphatic system is involved in PD, and that DTI-ALPS index may be a promising biomarker of the glymphatic system in PD patients.

In an article led by Butler et al., from Roche in Basel, Switzerland, in a case-control study data from a total of over 186,000 individuals, half of whom were diagnosed with Alzheimer's disease (AD), were examined to compare the prevalence of comorbidities between AD cases and individually-matched controls during the 5 years prior to diagnosis (or index date for controls). Comorbidities were also identified with a differential time-dependent prevalence trajectory prior to AD diagnosis. The authors found a greater comorbidity burden among those who later developed AD than in controls, and identified five main comorbidity clusters three of which contained comorbidities that increased in frequency over time in AD cases but not in controls during the 5-year period before AD diagnosis. These clusters may help in distinguishing AD cases and non-cases.

Kim et al., from the Department of Neurosurgery of the Hallym University College of Medicine, in Anyang, South Korea, examined the Korean National Health Insurance Database to investigate whether patients with Alzheimer's disease (AD) or Parkinson's disease (PD) were more likely to contract COVID-19 and experience worse outcomes. The study showed that patients with COVID-19 infection were more likely to have a pre-existing AD diagnosis or a PD diagnosis (although the association did not reach significance for PD). In addition, having AD (but not PD) was associated with higher COVID-19-related mortality and both diseases were associated with higher odds of severe COVID-19 infection. The findings underscore the importance of COVID-19 prevention measures and the need for further research in the risk and outcomes of COVID-19 infections in patients with AD and PD.

Soon et al. sought to determine the extent to which an imaging technique could potentially help guide management for patients undergoing investigation for normal pressure hydrocephalus. Decisions about the appropriateness of a ventricular shunt placement are often based on the extent to which the symptoms of gait impairment, incontinence, and cognitive impairment, respond to external lumbar drainage. Novel 3-dimensional linear indices describe both the directional expansion of the ventricles and describes the distribution of fluids across compartments. Using a validated modified Frailty Index, the authors determined the frailty status in 21 individuals with probable normal pressure hydrocephalus. The morphological 3-directional morphological indices derived from MR imaging were not adequate to predict which patients responded to CSF drainage. The study reinforces the importance that neither frailty status nor MR findings should be used to predict clinical responsiveness to shunting. However, there may be utility in using this imaging to differentiate patients with NPH from those with Alzheimer's and health controls.

The article by She et al. examined the prevalence and pattern of comorbidity among Chinese patients with first-ever ischemic stroke within 2 weeks of admission to the University Hospitals in Shandong, China. Comorbidity was present in more than 90% of the stroke- patients; and was significantly higher among women. These comorbidities clustered into three patterns namely degenerative-cardiopulmonary disease, heart-gastrointestinal-psychiatric disease; and metabolic-kidney disease. On further investigating the association of comorbidities and physical dependence and cognitive functions among acute phase stroke patients, She et al. identified that a higher number of comorbidities was associated with a greater likelihood of physical dependence and cognitive impairment. Almost all the disease clusters were positively associated with the physical dependence and poor cognition among these patients. The findings from the study highlight the high burden of co-morbidity among stroke patients and the relevance of identifying these diseases which are associated with the physical and cognitive function of the patients. The authors reinforce the need for longitudinal studies to generate stronger evidence for this association.

In a longitudinal study among Japanese older adults without Cardio Vascular Disease (CVD), Makino et al. examined the prospective associations of absolute CVD risk using the WHO region-specific risk estimation charts, with the incidence of cognitive impairment (CI). Within a median follow up period of 48 ± 2 months, the incidence of cognitive impairment in any domain was 13% in this population. Higher level of CVD risk at baseline was significantly associated with higher risk of any type of cognitive impairment. However, this association was different between the subtypes of CI. While the baseline CVD risk predicted the incidence of non-amnestic CI, it was not associated with amnestic CI. Through this article the authors demonstrate that CVD risk level -which can be easily obtained in clinical practice- is a valuable tool for subtype specific screening of dementia.

We expect that the series of articles published in the current Research Topic will further our understanding of multimorbidity within the Nervous System, and stimulate new experimental models to better understand the interaction of two or more simultaneous conditions, as well as the design of novel therapeutic approaches to Neurodegenerative Diseases.

## Author contributions

All authors listed have made a substantial, direct, and intellectual contribution to the work and approved it for publication.

## Conflict of interest

MV received funding from F. Hoffmann-La Roche Ltd. and Biogen and consulted for F. Hoffmann-La Roche Ltd., outside of this study; MV has equity ownership in Abbott Laboratories, Johnson and Johnson, Medtronic, Merck, AbbVie and Amgen. The remaining authors declare that the research was conducted in the absence of any commercial or financial relationships that could be construed as a potential conflict of interest.

## Publisher's note

All claims expressed in this article are solely those of the authors and do not necessarily represent those of their affiliated organizations, or those of the publisher, the editors and the reviewers. Any product that may be evaluated in this article, or claim that may be made by its manufacturer, is not guaranteed or endorsed by the publisher.
